# Technology-Assisted Self-Regulated English Language Learning: Associations With English Language Self-Efficacy, English Enjoyment, and Learning Outcomes

**DOI:** 10.3389/fpsyg.2020.558466

**Published:** 2021-01-05

**Authors:** Zhujun An, Chuang Wang, Siying Li, Zhengdong Gan, Hong Li

**Affiliations:** ^1^University of Macau, Taipa, China; ^2^Chongqing University of Education, Chongqing, China

**Keywords:** technology-assisted language learning, self-regulated learning, English language self-efficacy, English enjoyment, learning outcomes

## Abstract

This study investigated Chinese university students’ technology-assisted self-regulated learning (SRL) strategies and whether the technology-based SRL strategies mediated the associations between English language self-efficacy, English enjoyment, and learning outcomes. Data were collected from 525 undergraduate students in mainland China through three self-report questionnaires and the performance on an English language proficiency test. While students reported an overall moderate level of SRL strategies, they reported a high level of technology-based vocabulary learning strategies. A statistically significant positive relationship was noted between the use of technology-based SRL strategies and students’ English learning outcomes. English language self-efficacy and English language enjoyment were both related to technology-based SRL strategies. Furthermore, SRL strategies fully mediated the relationship between English enjoyment and English learning outcomes, but the association between English enjoyment and SRL strategies was only partially mediated by English language self-efficacy. Pedagogically, findings of this study suggest that training and instruction aimed at promotion of modern educational technology among students need to give attention to developing their strategic awareness of motivation regulation in optimizing effectiveness of their technology use in learning the target language.

## Introduction

The use of technology has received increasing recognition as a means capable of bridging formal and informal settings in the target language learning (Jung, 2015; Botero et al., 2018) and enabling students to actively and effectively use technology both inside and outside the classroom. There has been an increasingly large body of research on students’ use of technology for second or foreign language learning (e.g., Lee et al., 2016; Godwin-Jones, 2018; Lai et al., 2018; Su et al., 2018). These research studies have generally concentrated on students’ perceptions and evaluations of the suitability of technological devices for language learning, adoption of these technological devices in the classroom settings, and the factors that affect the effectiveness of language learning in classroom technology-using conditions (Winke and Goertler, 2008; Peters et al., 2009; Steel and Levy, 2013). For example, Winke and Goertler (2008) found that songs and movies were the most frequently used technologies and the ease of access was the strongest predictor of the frequency of technology use. Peters et al. (2009) also reported that listening to music and viewing video files were two of the most highly preferred activities. Recent research on technology-facilitated language learning, however, has been mostly laboratory and classroom experiments of technology applications in the formal educational contexts (e.g., Burston, 2015; Demouy et al., 2016; Kukulska-Hulme, 2016; Tseng and Yeh, 2019). Consequently, our knowledge and understanding of students’ self-regulated use of technology for target language learning is still limited. Aspects of technology-assisted language learning such as goal setting, motivation-regulation, and cognitive strategy use particularly in an English as a Foreign Language (EFL) context remains in need of further empirical inquiry. After all, in the course of learning a second or foreign language, learners are at the center of learning and play an instrumental role in shaping outcomes of their learning experiences. Key to this view of learner-centredness is self-regulation and learners taking the responsibility for their own learning (Holec, 1981). Nevertheless, what is lacking in recent research on technology-assisted language learning is a systematic examination of SRL strategies in technology-using conditions particularly in an EFL context. This study investigated Chinese university students’ technology-assisted self-regulated learning (SRL) strategies and whether the technology-based SRL strategies mediated the associations between English language self-efficacy, English enjoyment, and learning outcomes.

### Self-Regulated Learning

Self-regulated learning has been widely acknowledged to be learners’ systematic effort to manage and regulate their learning process in order to achieve particular learning goals (Pintrich, 2004; Zimmerman and Schunk, 2011). While different theoretical models provide different definitions of SRL, there is a consensus that SRL is a multifaceted construct containing cognitive, metacognitive, behavioral, and self-motivational aspects (Zimmerman and Schunk, 2001; Schunk and Zimmerman, 2012). According to Pintrich et al. (1991), cognitive strategies refer to the skills that learners use to process the information and knowledge when completing a task. They help students to construct, transform, and apply second language (L2) knowledge (Oxford, 2013). Metacognitive strategies refer to the skills students use to control and regulate the cognition and cognitive resources, which helps in goal setting, planning, monitoring, and evaluating their learning outcomes (Winne, 2011). Social-behavioral strategies, as a key aspect of self-regulation, involve learners’ control over their learning behavior under the influence of contextual aspects (Zimmerman, 2011). Finally, motivational strategies refer to the procedure or thoughts students applied intentionally to sustain or increase their interest to engage in a task (Wolters, 1999). Note that in various models of SRL in the literature (e.g., Butler and Winne, 1995; Nicol and Macfarlane-Dick, 2006), self-regulated learners are depicted as being capable of controlling over the cognitive, emotional, motivational, and behavioral aspects of learning (Zimmerman and Schunk, 2011). Research also shows that those more effective at self-regulation use a broader repertoire of learning strategies and persist longer in the face of adversity compared to their less self-regulated counterparts (Pajares, 2009).

### Self-Regulated Language Learning in Technology-Using Conditions

Benson (2001) described two important categories of learning resources: traditional learning resources (e.g., reference and course books) and resources provided by modern educational technology (e.g., information communication technology applications). According to Benson, self-regulation is manifested not only in the active regulation of learning strategies but also in the management of different kinds of learning resources. Self-regulated language learning in technology-using conditions has thus often been described as being characterized by learners developing learning strategies such as planning and resource management, and reflecting on as well as evaluating their learning behavior and outcomes (Carneiro et al., 2007). As such, technology-based self-regulated English learning (SRL) strategies refer to specific actions taken by the learners to learn English or to enhance their English learning in technology-using conditions. A large quantity of technology-assisted SRL strategies were identified in previous studies conducted in a variety of research contexts, such as consulting online dictionaries, using translation software, reading texts on the computer, searching the web for information, listening to the radio, exploring cultural knowledge on YouTube and so on (Steel and Levy, 2013; Lai et al., 2018; An et al., 2020; Wang and Chen, 2020).

A number of studies also provide evidence that technology-assisted SRL strategies impact the improvement of learning performance. Chang and Chang (2014) explored Taiwanese college students’ listening comprehension strategies on the platform of YouTube, with data collected by Oxford’s (1990) Strategy of Inventory for Language Learning (SILL). They found that students performed significantly better on listening comprehension tests after the metacognitive instructional process. Bekleyen and Hayta (2015) investigated the role of mobile phone technology in the employment of language learning strategies among Turkish undergraduate students. Their study employed a self-designed questionnaire to collect data on students’ language learning strategies, which was also based on Oxford’s (1990) classification of the language learning strategies. Their results show that different types of mobile phone-assisted language learning strategies are helpful in improving students’ English proficiency. Nevertheless, constrained by the adoption of Oxford’s (1990) classification of the language learning strategies, these studies largely focused on students’ use of cognitive and metacognitive strategies. Furthermore, self-regulation is context- and situation-specific, which means that measurement of technology-based self-regulated language learning should be domain-specific (Wang and Zhan, 2020).

Somewhat unlike the studies reviewed above, Lai and Gu (2011) examined how language learners relied on their metacognitive knowledge to regulate different aspects of their language learning experience, and further identified various factors that affected the participants’ selective use of technology for language learning. Encouraging as Lai and her associates’ findings were, their study did not include and report cognitive strategies learners used in technology-assisted second language learning contexts. Given the potential technology opens up for language learning, we believe that knowledge of what technology-based cognitive learning strategies students prefer and what strategies were possibly omitted in the previous research will be useful in designing student training programs. We therefore suggest that technology-assisted strategic language learning as a process be examined from a multidimensional perspective that includes an understanding of cognitive, metacognitive, social, and motivational components, and an understanding of how they interact with individual factors and learning achievement.

### Self-Efficacy

Self-efficacy refers to individuals’ personal evaluations of their capability of accomplishing a particular task (Schunk et al., 2008). According to Bandura (2006), efficacy beliefs influence the courses of action people choose to pursue, the challenges and goals they set for themselves and their commitment to them, how much effort they put forth in given endeavors, and the outcomes they expect their efforts to produce. Social cognitive theorists have emphasized the role of self-efficacy beliefs in SRL as predictors of academic performance as self-efficacy beliefs can be modified in school practice to promote better academic performance (Zuffianò et al., 2013). The basic presumption as to why self-efficacy has an effect on students’ SRL is that when students experience feelings of worth and a perception of improved capability, students are likely to perform better and therefore to experience successful performance (Panadero et al., 2017). For example, in Bassi et al.’s (2007) study, high self-efficacy students were found to report higher academic aspirations and pursuits than low self-efficacy students, and spend more time in homework and primarily associate learning activities with optimal experience. Self-efficacy beliefs have therefore been acknowledged to fulfill a significant role in understanding the academic lives of students as it influences their motivation, affect, and behaviors (Bandura, 2006). While there is prolific research on self-efficacy in the general education field, it is only within the past two decades that self-efficacy has been attracting researchers’ attention in the field of L2 acquisition. Similarly, in a study of graduate pre-service teachers’ language learning strategies and language self-efficacy in Malaysia, Wong (2005) reported that high self-efficacy pre-service teachers reported more frequent use of more language learning strategies than did low self-efficacy pre-service teachers. A study of the influence of self-efficacy and other motivational self-beliefs on the achievement among college intermediate French students also revealed that self-efficacy for self-regulation was the most significant predictor of intermediate French language achievement, and that students who perceived themselves as capable of using effective metacognitive strategies to monitor their academic work time effectively were more apt to experience academic success in intermediate French (Mills et al., 2007).

Note that more recently, a number of L2 studies tended to develop new self-report self-efficacy measurements to investigate the role of self-efficacy in the L2 learning process. For example, to address the need for valid and reliable tools to assess ESL learners’ self-efficacy, Wang et al. (2013) developed the English Self-Efficacy Questionnaire to measure English self-efficacy in the following four areas: (1) English listening; (2) English speaking); (3) reading; and (4) writing. Subsequent Confirmatory factory analysis (CFA) with data from Chinese university students confirmed a second-order common factor with these four first-order latent constructs: English listening, English speaking, English reading, and English writing. To date, studies that adopted the English Self-Efficacy Questionnaire showed that students’ English language self-efficacy influenced their use SRL (e.g., Wang and Bai, 2017). English language self-efficacy was also found to positively influence students’ feedback preferences and behavior in academic English course settings (Bai and Wang, 2020; Gan et al., 2020). In the existing studies on learners’ use of information and communication technologies, however, self-efficacy tends to be operationalized as students’ confidence in their ability to select appropriate technological solutions and utilize the chosen technologies effectively to meet learning needs (Lai and Gu, 2011). To the best of our knowledge, the role of the learners’ English language self-efficacy in determining their selection and utilization of technology for English learning and enhancement has not been researched.

### Foreign Language Enjoyment

Positive emotions, such as enjoyment, pride, and flow, have been regarded as being efficient in facilitating learning (MacIntyre and Gregersen, 2012; Lake, 2013). Among the positive emotions, enjoyment has been recognized as a most typical positive emotion experienced by foreign language learners and has received increasing attention from researchers in the field of educational psychology (e.g., Csikszentmihalyi, 1990; Dewaele and MacIntyre, 2016, Dewaele et al., 2017; Li et al., 2018). Enjoyment was a sense of satisfaction and reward that generated from activities or the achievement of activities (Ainley and Hidi, 2014). In the literature of educational psychology, enjoyment is often defined as a positive psychological state coming from the efforts by the person who stretches beyond himself to accomplish something challenging or difficult (Csikszentmihalyi, 1990). In the area of English language education, English enjoyment refers to students’ liking for learning English as a foreign language. Specifically, in the foreign language learning context, experiencing enjoyment involves concentration, clearing goals and immediate feedback that can help learners build resources (Li et al., 2018). Dewaele and MacIntyre (2016) observed that individual learners who were more proficient than their classmates and who eventually reached a higher level proficiency in the target language demonstrated a significantly higher level of enjoyment than their peers. Foreign language enjoyment was also positively associated with academic achievement by promoting psychological resiliency, relieving negative arousal, and broadening learners’ instant thought-action repertoires (Ryan et al., 1990; Lai et al., 2018; Piniel and Albert, 2018). In the literature on students’ use of technology for language learning, while there were various accounts of language learners actively engaging in self-initiated learning activities, it was also frequently reported that there was little and limited use of technologies for language improvement particularly outside the classroom, despite the frequent use of a wide repertoire of technologies for entertainment or infotainment (Lai and Gu, 2011). Such little and limited use of technologies for real language learning purposes is often attributed to a lack of intrinsic interest or enjoyment toward the target language the students are learning. Nevertheless, this assumption has not been empirically well-tested yet.

Clearly, it can be concluded from the above review that while there has been an attempt to integrate learning strategies with elements of SRL and metacognition in the context of technology supported language learning, the literature on the application of self-regulation in technology-supported second language learning is still fairly limited. Although the importance of the role of the strategic and motivational factors in first and second language contexts has been well documented, how these factors function in relation to students’ learning achievement in the context of technology use for self-regulated language learning has been under-researched.

### Associations Among Technology-Based SRL Strategies, English Language Self-Efficacy, English Enjoyment, and Learning Outcomes

According to the Expectancy-Value Theory (EVT) (Wigfield and Eccles, 2000; Eccles, 2009), students’ expectancies and values play significant roles in their academic learning. Specifically, individual learners’ expectancies for success and their subjective values (i.e., attainment, intrinsic value, extrinsic utility, and cost) are assumed to directly influence their educational and behavioral choices. Expectancies and values also directly influence learners’ performance, effort, and achievement (Wigfield and Eccles, 2000). Expectancies for success are usually similar to Bandura’s efficacy expectation in his discussion of self-efficacy, referring to individuals’ beliefs about how well they will do on upcoming tasks, either in the immediate or longer term future. Subjective task values include dimension such as intrinsic interest (e.g., enjoyment) and utility (e.g., usefulness). From a social cognitive theory perspective, self-efficacy beliefs are shaped through a blend of personal experiences, vicarious experiences, social persuasion, and interpretations of physical and emotional condition (Bandura, 1994; Walker and Greene, 2009), and emotional arousal is an essential source of the self-efficacy formation (Bandura, 1997). As such, the sense of enjoyment experienced in relation to a given task situation should thus help people feel confident about their ability to successfully organize and execute a given course of action to solve a problem or accomplish a task (Bandura, 1994). Consequently, EVT and Bandura’s self-efficacy theory serve as the major conceptual framework supporting the existence of the chain mediation process, i.e., enjoyment→self-efficacy→SRL→learning outcomes.

Empirical studies have also consistently shown that learning enjoyment and self-efficacy are critical determinants of learners’ SRL strategy use and learning achievement. For example, Kim et al. (2015) study revealed that students with high levels of self-efficacy are more likely to devote efforts and use various SRL strategies during the process of learning. Bai and Guo (2019) also found that learning interest, self-efficacy, and growth mindset can significantly promote learners’ SRL strategy use and impact their learning achievement. In Bai and Wang’s (2020) study, English self-efficacy and intrinsic value were found to be positive predictors of students’ self-regulated language learning strategies which in turn contribute to the improvement of English learning outcomes. Similarly, in their study of the role of the motivation variables of self-efficacy, enjoyment, and learning goal orientation in predicting the use of Web-based information systems, Yi and Hwang (2003) found that enjoyment significantly predicted students’ self-efficacy. More recently, Hong et al.’s (2017) study on the interrelatedness between intrinsic motivation, online learning self-efficacy, flow experience and students’ learning progress also showed that increasing intrinsic motivation (e.g., enjoyment) for language learning increases online learning self-efficacy and flow experience. These studies thus provide empirical evidence that foreshadows the mediation process, i.e., enjoyment→self-efficacy→SRL→learning outcomes. It needs to be pointed out, however, that previous studies that have investigated the associations among enjoyment, self-efficacy, SRL strategies, and performance have rarely included these variables in one model in relation to students’ learning achievement in the language context.

### Present Research

**FIGURE 1:**
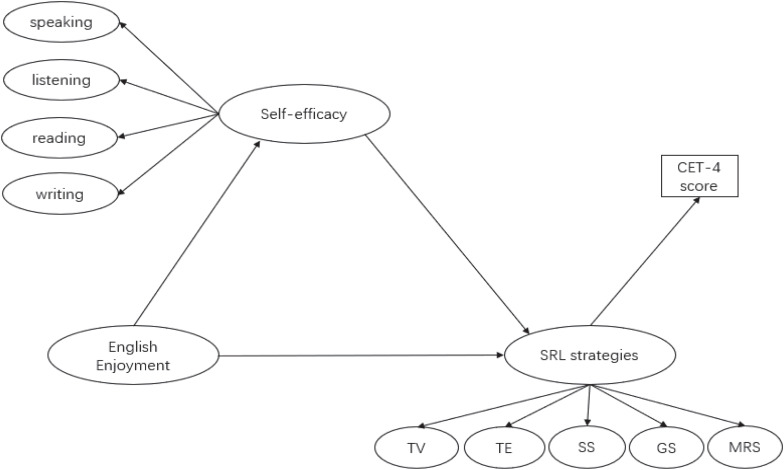
Hypothesized structural model of the relationships among technology-based SRL strategies, English language self-efficacy, English enjoyment, and English learning outcomes (i.e., CET-4 score). SRL strategies, technology-based self-regulated English learning strategies; MRS, motivational regulation strategies; GS, goal setting and planning; SS, social strategies; TE, technology-based English song and movie learning; TV, technology-based vocabulary learning.

Guided by the EVT (Wigfield and Eccles, 2000; Eccles, 2009), this study focused on examining in Chinese university EFL undergraduate students the interplay between English language self-efficacy, English enjoyment, technology-based SRL strategies, and their English learning outcomes. The students’ English learning outcomes in this study refer to their score they obtained in the National College English Test-Band 4 (CET-4). In line with the expectancy and value theory perspective and empirical studies discussed above, the research model in Figure 1 proposes that both English language self-efficacy and English enjoyment influence technology-based SRL strategies which in turn influence students’ English learning outcomes, and that English enjoyment is hypothesized to predict students’ English language self-efficacy.

Five research questions were thus addressed in this study:

1. What are the reported types and frequencies of Chinese university EFL students’ technology-based SRL strategies?
2. How do different types of technology-based SRL strategies contribute to EFL students’ English learning outcomes?
3. How does students’ English language self-efficacy associate with technology-based SRL strategies?
4. To what extent does English enjoyment correlate with technology-based SRL strategies?
5. What are the relationships among students’ English language self-efficacy, English enjoyment, technology-based SRL strategies, and English learning outcomes?

In order to answer the research questions, and based on the analysis of the literature, the following hypotheses are established accordingly:

H1: Different types of technology-based SRL strategies display differential associations with English learning outcomes.
H2: English language self-efficacy positively predicts technology-based SRL strategies.
H3: English enjoyment positively predicts technology-based SRL strategies.
H4: English enjoyment positively predicts students’ English language self-efficacy.
H5: There is a significant positive relationship between the use of technology-based SRL

strategies and English learning outcomes.

## Materials and Methods

### Participants

A total of 525 undergraduate students forming a volunteer multidisciplinary sample from a university in Northern China were recruited. All participants were first-year and second-year undergraduate students, whose age ranged from 17 to 25 (*M* = 20.50, *SD* = 7.97). Most participants were majoring in accounting, finance, auditing, and management. Thus, female students (*n* = 377) outnumbered male students (*n* = 148). At the time of data collection, all the students had received formal English education for over 6 years and experienced College Entrance Examination in mainland China. English is a compulsory course for first-year and second-year students in the university where English teachers and students meet in class for 3–4.5 h per week in classrooms. All students in the university were required to pass CET4 before graduation.

### Procedure for Data Collection

The first author contacted the College English course instructors at the university about their willingness to include their students in this survey. Ethical approval was obtained from the university before the study was carried out. Those instructors who were willing to include their students in the survey were then asked to help distribute the online survey weblink to their students by any means that they normally used to contact their students. All participants were informed that their participation was voluntary and that they could withdraw from the study at any time. The participants were also informed that their responses to our survey would be kept confidential and that all data collected would be securely stored in the research center, and would only be used for research purposes. They completed the online survey outside the classroom on the platform of Wenjuanxing with no time limit. The average time the participants used in completing the online survey was 230.01 s.

### Instruments

#### Technology-Based Self-Regulated English Learning Strategies Questionnaire

The technology-based SRL strategies questionnaire (TSRLSQ) was designed to assess participants’ SRL strategy use in technology-using language learning conditions. Items in the questionnaire originated from three major sources: (a) constructs related to self-regulation or SRL strategies (e.g., cognitive, metacognitive, behavioral, and motivational regulation strategies) outlined by Pintrich and De Groot (1990); Zimmerman (2011), and Oxford (2013); (b) focus group interviews with 15 undergraduate students about the strategies they used when learning English in technology-using conditions; (c) existing instruments assessing students’ technology use in language learning or SRL strategies (Barnard et al., 2009; Toffoli and Sockett, 2010; Lai et al., 2012; Lee et al., 2016). These processes resulted in a generation of 36 initial items. Two professional academics who were familiar with SRL strategies with technology were invited to assess the face and content validity of each item. An item was retained if both the two academics agreed that it is appropriate to be used to evaluate students’ technology-based SRL in the Chinese EFL context. As a result of this validation process, 30 items were retained and slight modifications were made in the wordings of a few items in light of the two academics’ comments.

The 30-item questionnaire in which items were scored on a 7-point Likert scale, ranging from 1 (not at all true of me) to 7 (very true of me), was then piloted to a sample of 155 university EFL students in China. Exploratory factor analyses (EFA) was conducted to examine the underlying factor structure of the TSRLSQ. The Bartlett’s spherical test provided a significant chi-square value of 3,069.86 (*p* < 0.001), and the Kaiser-Meyer-Olkin (KMO) statistic was 0.92 exceeding the minimum adequacy value of 0.50 (Tabachnick and Fidell, 2007), meaning that the data were suitable for structure detection (Kaiser, 1958). The Laiser’s eigenvalues-greater-than-1.00 criterion (Kaiser, 1960) and the scree plot (Raubenheimer, 2004) extracted a five-factor structure with 26 items, accounting for 69.62% of the total variance: (1) motivational regulation strategies (9 items); (2) goal setting and learning evaluation (5 items); (3) social strategies (4 items); (4) technology-based English song and movie learning (5 items); and (5) technology-based vocabulary learning (3 items).

The above 26-item five-factor structure of Chinese EFL students’ TSRLSQ was cross-validated in the present study with a sample of 525 undergraduate students, suggesting an overall acceptable model fit with *X*^2^ = 1018.14 (df = 281, *p* < 0.001); CFI = 0.91; TLI = 0.90; SRMR = 0.048; RMSEA = 0.07). Standardized factor loadings for CFA of the TSRLSQ ranged from 0.33 to 0.87. A scale analysis of the total 26 items in TSRLSQ revealed high internal consistency (Cronbach’s alpha = 0.95). The Cronbach’s alpha coefficients for the five factors were: 0.91 for motivational regulation strategies, 0.85 for goal setting and learning evaluation, 0.87 for social strategies, 0.86 for technology-based English song and movie learning, and 0.68 for technology-based vocabulary learning.

#### English Language Self-Efficacy Questionnaire

The 16 items in the English language self-efficacy Questionnaire in this study were adapted from Author (2013) that measures students’ English language self-efficacy in terms of four different domains: speaking, listening, reading, and writing, using a scale from 1 (not at all true of me) to 7 (very true of me). In the current study, CFA was also performed to confirm the four factor domains, and satisfying model fit indices were found with *X*^2^ = 436.49 (df = 96, *p* < 0.001), CFI = 0.96, TLI = 0.95, RMSEA = 0.08, SRMR = 0.03. Standardized factor loadings for CFA ranged from 0.75 to 0.93. A Cronbach’s alpha coefficient of 0.97 was found for the total items in the questionnaire. Furthermore, the Cronbach’s alpha for the four domains of English language self-efficacy were:0.92 for speaking, 0.90 for listening, 0.93 for reading, and 0.93 for writing.

#### English Enjoyment Questionnaire

The English Enjoyment Questionnaire in this study contains seven items adapted from the foreign language enjoyment questionnaire used in Dewaele and MacIntyre (2014) and Li et al. (2018), measuring classroom-based foreign language enjoyment. This questionnaire also adopts a 7-point Likert scale, ranging from 1 (not at all true of me) to 7 (very true of me). CFA was also performed to confirm the one factor structure of the questionnaire and good model fits were found with χ^2^ = 20.10 (df = 8, *p* < 0.001), CFI = 0.99, TLI = 0.98, SRMR = 0.03. The Cronbach’s α for the single one factor of the questionnaire was 0.84.

#### College English Test-Band 4 (CET-4)

In our present study, CET-4 was used to measure the participants’ English learning outcomes. As the most influential English proficiency test throughout colleges in China (Jin, 2008), the CET is administered by the National College English Testing Committee on behalf of the Chinese Ministry of Education (Zheng and Cheng, 2008). CET aims to provide an objective evaluation of a student’s overall English proficiency and positively affect EFL teaching at the tertiary level in China (State Education Commission, 1986). The CET-4 test-takers are undergraduate students in China except English majors. Each test takes 125 min to complete, with the total score of 710, containing four parts: writing (15%), listening comprehension (35%), reading comprehension (35%), and translation (15%) (Zheng and Cheng, 2008). The CET has been subjected to rigorous validation processes to ensure its high quality as an assessment tool for undergraduates (Yang and Weir, 1998).

### Data Analysis

Descriptive statistics (e.g., mean, standard deviation, and internal consistencies) for technology-based SRL strategies were calculated to answer the first research question. To answer the second research question regarding how students’ self-regulated technology-using English learning strategies were related to English learning achievement, five linear regression analyses (Models 1A–E) and one multiple regression analysis (Model 2) were run with the five types of strategies as predictors and the learning achievement as the outcome variable. For Models 1A–E, motivational regulation strategies, goal setting, and learning evaluation, social strategies, technology-based English song and movie learning, and technology-based vocabulary learning were regarded as predictors, respectively, in each model. For the one multiple regression analysis, the overall technology-based SRL strategy use was considered to be the predictor. This step was necessary, because the significance of exploring self-regulated technology-using English learning strategies is to see whether they are helpful for learning achievement. Adjusted R-squared values were reported as effect sizes.

To answer the third research question, Pearson product-moment correlation was carried out to assess the relationships between English self-efficacy, English enjoyment, and students’ SRL strategies in technology-using conditions. All the analyses were performed with IBM SPSS 24.0 (SPSS Inc., Chicago, IL, United States). Finally, the data were subjected to AMOS using structural equation models (SEM) to investigate the structural relationships between SRL strategies, English language self-efficacy, English enjoyment, and English learning outcomes. The following model fit indices were used for evaluating the model fit (Hu and Bentler, 1999): the chi-square statistic (χ2) and its degrees of freedom (*df*), along with the associated *p-*value; the Comparative Fit Index (CFI) (a value equal to or greater than 0.90 indicates acceptable model fit); Tucker-Lewis Index (TLI) (a value equal to or greater than 0.90 indicates acceptable model fit); the Root Mean Square Error of Approximation (RMSEA) (a value between 0.05–0.08 indicates good fit); and the Standardized Root Mean-square Residual (SRMR) (a value less than 0.08 indicates good fit).

## Results

### What Are the Reported Types and Frequencies of Chinese University EFL Students’ Technology-Based Self-Regulated English Learning Strategies?

Confirmatory factory analysis analysis in this study confirmed the five factor structure of the TSRLSQ: (1) motivational regulation strategies; (2) goal setting and learning evaluation; (3) social strategies; (4) technology-based English song and movie learning; and (5) technology-based vocabulary learning. The means, standard deviations, and Cronbach’s alpha reliability coefficients for the five types of SRL strategies were presented in Table 1. As suggested by Oxford (1990), in the case of a seven-point Liker scale, a variable mean in the range of 4.9–7.0 is usually considered to be high level, 3.5–4.8 medium level, and 1.0–3.4 low level (Guo and Wei, 2019). As such, among the five types of SRL strategies, technology-based vocabulary learning is the only type of strategies that was reported to be highly frequently used (*M* = 5.26, *SD* = 1.21), whereas the other four types of strategies, i.e., motivational regulation strategies (*M* = 4.36, *SD* = 1.24), English song and movie learning strategies (*M* = 4.11, *SD* = 1.34), goal setting and learning evaluation strategies (*M* = 3.95, *SD* = 1.30), and social strategies (*M* = 3.77, *SD* = 1.45), were reported to be used at a medium level.

**TABLE 1:** Correlation coefficients between the latent variables.

	Mean	SD	Cronbach’s alpha	1	2	3	4	5	6	7	8	9	10	11	12	13
SRL	4.25	1.13	0.95	1.00												
MRS	4.36	1.24	0.91	0.95***	1.00											
GS	3.95	1.30	0.85	0.88***	0.77***	1.00										
SS	3.77	1.45	0.87	0.85***	0.78***	0.69***	1.00									
TE	4.11	1.34	0.86	0.88***	0.78***	0.69***	0.70***	1.00								
TV	5.26	1.21	0.68	0.62***	0.52***	0.53***	0.33***	0.49***	1.00							
Efficacy	3.36	1.33	0.97	0.63***	0.60***	0.57***	0.58***	0.58***	0.24***	1.00						
Speaking	3.34	1.46	0.92	0.60***	0.57***	0.53***	0.58***	0.55***	0.20***	0.92***	1.00					
Listening	3.34	1.39	0.9	0.59***	0.57***	0.53***	0.53***	0.55***	0.21***	0.94***	0.83***	1.00				
Reading	3.41	1.42	0.93	0.58***	0.55***	0.53***	0.49***	0.53***	0.25***	0.93***	0.77***	0.84***	1.00			
Writing	3.35	1.44	0.93	0.58***	0.55***	0.52***	0.54***	0.53***	0.25***	0.93***	0.80***	0.81***	0.85***	1.00		
Enjoyment	4.76	1.12	0.84	0.64***	0.61***	0.55***	0.51***	0.57***	0.41***	0.61***	0.60***	0.55***	0.56***	0.55***	1.00	
CET4	402.80	54.40		0.23***	0.24***	0.20***	0.18***	0.21***	0.08	0.38***	0.37***	0.31***	0.37***	0.36***	0.32***	1.00

***p < 0.001. MRS, motivational regulation strategies; GS, goal setting and learning evaluation; SS, social strategies; TE, technology-based English song and movie learning; TV, technology-based vocabulary learning.

The correlations among different types of technology-based SRL strategies, English language self-efficacy, English enjoyment, and learning achievement were also listed in Table 1. The results showed that EFL students’ technology-based SRL strategies were positively correlated with each other (rs = 0.33–0.95, ps < 0.001). Furthermore, different types of technology-based SRL strategies, English language self-efficacy, and English enjoyment had positive correlations with learning achievement (rs = 0.18–0.38, *p* < 0.001), except technology-based vocabulary learning strategy (*r* = 0.08, *p* > 0.05).

### How Do Different Types of Technology-Based SRL Strategies Contribute to EFL Students’ English Learning Outcomes?

Regression analyses of technology-based SRL strategies as predictors of English learning outcomes showed that motivational regulation strategies (β = 0.24, *p* < 0.001, Δ*R*^2^ = 0.06) (see model 1A in Table 2), goal setting and learning evaluation (β = 0.20, *p* < 0.001, Δ*R*^2^ = 0.04) (see model 1B in Table 2), social strategies (β = 0.18, *p* < 0.001, Δ*R*^2^ = 0.03) (see model 1C in Table 2), as well as English song and movie learning (β = 0.21, *p* < 0.001, Δ*R*^2^ = 0.04) (see model 1D in Table 2) were statistically significantly associated with students’ English learning outcomes, whereas technology-based vocabulary learning (β = 0.08, *p* > 0.05, Δ*R*^2^ = 0.004) was not (see model 1E in Table 2). Additionally, when all five types of technology-based SRL strategies entered the equation simultaneously, it turned out that motivational regulation strategies (β = 0.24, *p* = 0.006, Δ*R*^2^ = 0.06) was the only significant predictor of the CET-4 score (see model 2 in Table 2).

**TABLE 2:** Regression models of technology-based SRL strategies as predictors for English learning outcomes.

		English learning outcomes		
Model		Δ*R*^2^	SE(B)	β
1A	Motivational regulation strategies	0.06	1.86 (10.64)	0.24***
1B	Goal-setting and learning evaluation	0.04	1.79 (8.40)	0.20***
1C	Social strategies	0.03	1.62 (6.76)	0.18***
1D	Technology-based English song and movie learning	0.04	1.74 (8.44)	0.21***
1E	Technology-Based vocabulary learning	0.004	1.96 (3.37)	0.08
2	Motivational regulation strategies	0.06	3.84 (10.68)	0.24**
	Goal-setting and learning evaluation		3.00 (2.63)	0.06
	Social strategies		2.71 (−2.56)	−0.07
	Technology-based English song and movie learning		2.93 (2.73)	0.07
	Technology-based vocabulary learning		2.36 (−4.26)	−0.10

**p < 0.01; ***p < 0.001.

### To What Extent Do Students’ English Language Self-Efficacy and English Enjoyment Correlate With Different Types of Technology-Based SRL Strategies?

Pearson product-moment correlation (Table 1) between English language self-efficacy, English enjoyment, and students’ technology-based SRL strategies showed that English language self-efficacy (*r* = 0.63, *p* < 0.001) and English enjoyment (*r* = 0.64, *p* < 0.001) positively correlated with the overall technology-based SRL strategies. Specifically, all the four domains of English self-efficacy (speaking, listening, reading, and writing) significantly positively correlated with the five different types of technology-based SRL strategies, among which technology-based vocabulary learning strategies had the weakest correlations with both the overall English language self-efficacy and the four different domains of English language self-efficacy (rs = 0.20–0.25, ps < 0.001). English enjoyment also positively correlated with the five different types of technology-based SRL strategies (rs = 0.41–0.61, ps < 0.001).

### What Are the Relationships Among Students’ English Language Self-Efficacy, English Enjoyment, Technology-Based SRL Strategies, and English Learning Outcomes?

**FIGURE 2:**
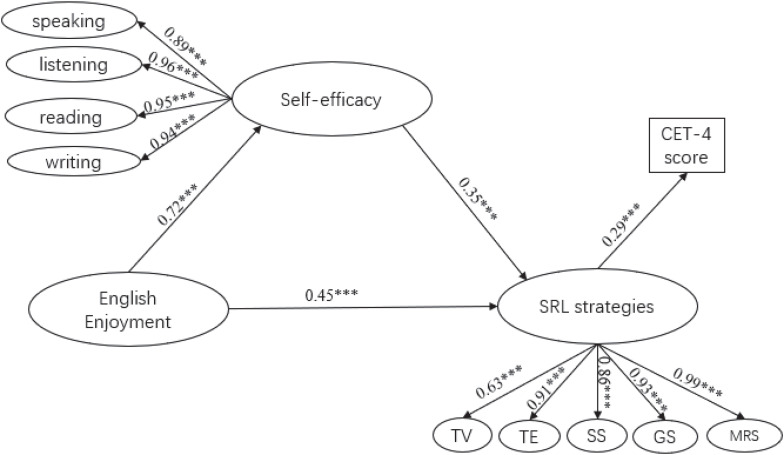
Structural model of the relationships between technology-based SRL strategies, English language self-efficacy, English enjoyment, and English learning outcomes (i.e., CET-4). ^∗∗∗^*p* < 0.001. SRL strategies, technology-based self-regulated English learning strategies; MRS, motivational regulation strategies; GS, goal setting and planning; SS, social strategies; TE, technology-based English song and movie learning; TV, technology-based vocabulary learning.

Structural equation models model suggested that the data fit the hypothesized model, X^2^ = 2894.97 (*df* = 1143, *p* < 0.001); CFI = 0.90; TLI = 0.90; RMSEA = 0.05; SRMR = 0.06 (see Figure 2). The completely standardized parameter estimates of the significant correlations among the four variables are presented in Figure 2. Consistent with the finding of regression analyses and Pearson correlation analyses (Table 1), SRL strategies were statistically significantly associated with students’ English learning outcomes (β = 0.29, *p* < 0.001). English language self-efficacy was statistically significantly linked with SRL strategies (β = 0.35, *p* < 0.001), and English enjoyment was statistically significantly associated with the use of SRL strategies (β = 0.45, *p* < 0.001). Moreover, the path from English enjoyment to English language self-efficacy was statistically significant (*p* < 0.001), with a standardized loading of 0.72. These variables explained 6.9% of the variance in EFL undergraduate students’ English learning outcomes.

The mediating effect of English language self-efficacy was tested using bootstrapping approach. As shown in Table 3, the indirect effect of English enjoyment on technology-based SRL strategies via English language self-efficacy was equal to the product of the coefficients for each of the paths in the mediation chains (i.e., 0.72^∗^0.35 = 0.25). The 95% bias-corrected confidence interval for the mediated effect was between 0.17 and 0.34, with a *p*-value at 0.001 for the two-tailed significance test and the standard error at 0.04. The total effect of English enjoyment on technology-based SRL strategy use was 0.25 + 0.45 = 0.70. After controlling for English language self-efficacy, the direct relationship between English enjoyment and SRL strategies was also significant (β = 0.45, *p* < 0.01). Therefore, the effect of English enjoyment on technology-based SRL strategies was partially mediated by English language self-efficacy.

**TABLE 3:** Standardized direct, indirect, and total effects for structural model.

Predicted variable	Predictor variable	Direct effect	Indirect effect	Total effect
Learning outcomes	Technology-based SRL	0.29**		0.29**
	English self-efficacy		0.10**	0.10**
	English enjoyment		0.20**	0.20**
Technology-based SRL	English self-efficacy	0.35**		0.35**
	English enjoyment	0.45**	0.25**	0.70**
English self-efficacy	English enjoyment	0.72**		0.72**

**p < *0.01*.

The indirect effect of English language self-efficacy on English learning outcomes through technology-based SRL strategies was equal to 0.35^∗^0.29 = 0.10. The 95% bias-corrected confidence interval for the mediated effect was between 0.06 and 0.15, with a *p*-value at 0.001 for the two-tailed significance test and the standard error at 0.02. In addition, the indirect effect of English enjoyment on English learning outcomes via technology-based SRL strategies was 0.45^∗^0.29 = 0.13. The indirect effect of English enjoyment on English learning outcomes through English language self-efficacy and technology-based SRL strategies was 0.72^∗^0.35^∗^0.29 = 0.07. Hence the total effect of English enjoyment on English learning outcomes was equal to 0.13 + 0.07 = 0.20. The 95% bias-corrected confidence interval for the mediated effect was between 0.13 and 0.28, with a *p*-value at 0.002 for the two-tailed significance test and the standard error at 0.04.

## Discussion

One shortcoming of the previous studies on technology-assisted language learning is that lack of domain- and situation-specific instruments might result in failure to capture patterns of learning strategies that are idiosyncratic to technology-using conditions particularly in an EFL context. Building on SRL research in educational psychology and computer-assisted L2 learning research, this study investigated Chinese university students’ technology-assisted SRL strategies in relation to their English language self-efficacy, English enjoyment, and English learning outcomes. The study has confirmed five types of English learning strategies in technology-using conditions among Chinese university EFL students: (1) motivational regulation strategies; (2) goal setting and learning evaluation; (3) social strategies; (4) technology-based English song and movie learning; and (5) technology-based vocabulary learning. We concur with the argument that understanding the SRL strategies that students use has implications for classroom-based language teaching and learning (Zimmerman and Schunk, 2011). For example, technology-based vocabulary learning was reported to be the most frequently used strategy in this study; this strategy, however, unlike other types of SRL strategies, was found to have no positive impact on students’ English learning outcomes. In our communication with some study participants during the interviews in the course of questionnaire construction, we noted that learning English vocabulary through use of lexical apps on mobile phones was prevalent among the students. Given the results of this study, there is a pressing need to engage students in a variety of more meaningful vocabulary learning activities both inside and outside the classroom.

In this study, technology-based social strategies were reported to be the least frequently used ones among all five SRL strategies, which was somewhat unexpected because one of the great educational potentials of technology-assisted language learning environments is that they provide learners with interaction opportunities and easy access to authentic language input via communicating with native speakers (Golonka et al., 2014). One possible explanation might be that the students in this study were required to take CET-4 before graduation, which usually results in tons of mechanical CET-4 drilling exercises rather than social communicative learning activities for the students. Consequently, some students might believe their major goal of learning English is to pass the CET-4, and therefore, their technology-based English learning might be limited to lexical app-based words memorization and practice of multiple choice type of questions. An important implication of this result is thus that students should be encouraged to participate in more social language learning activities such as cooperative learning. Since teachers play an important role in engaging students in social or experiential learning activities, there is a pressing need to integrate communicative approach into the EFL curriculum so as to reduce the teacher prolonged control of the classroom and to maximize student responsibility and involvement in the social learning activities.

The regression analyses and SEM analyses in this study showed a positive relationship between technology-based SRL strategies and students’ English learning outcomes, supporting Hypothesis 5. This result is congruent with prior research findings on the positive relationship between SRL strategies and academic achievement in second language acquisition (e.g., Xiao and Yang, 2019; Bai and Wang, 2020; Teng and Zhang, 2020). An important pedagogical implication of this result is that teachers can raise students’ awareness of the importance of technology-based SRL strategies, introduce to them a wide variety of learning strategies, and model the use of these strategies in the classroom. In particular, the results of regression analyses highlight the importance of motivational regulation strategies as it is the only significant and positive predictor of CET-4 scores among the five types of technology-based SRL strategies. This evidenced the differential associations different types of technology-based SRL strategies display with English learning outcomes, supporting Hypothesis 1. Our result echoes findings of previous research on the vital role of motivational regulation strategies in students’ learning achievement (Wolters, 2003; Teng and Zhang, 2016) and classroom performance (Wolters, 1999). Note that the current TESOL Technology Standards for Language Learners (Healey et al., 2018) are more often focused on decontextualized knowledge and skills in technology use and evaluation of technology-based tools as aids in the development of their language learning competence with little regard to the role of motivation-regulation factors that are critical to sustaining students’ use of technology, and that impact on students’ learning outcomes. This study suggests that although knowledge and skills in using various technological tools are important, it is equally important to empower students, as agents, to develop and increase their strategic awareness of motivational regulation in optimizing effectiveness of their technology use in the target language learning. As teachers occupy a central role in promoting technology-assisted language learning among students, it is imperative to provide teachers with professional training to enrich their knowledge and skills in the utilization of motivational regulation strategies.

Furthermore, the SEM analyses in this study showed that technology-based SRL strategies mediated the relationship between English enjoyment and English learning outcomes. The findings indicated that students who enjoy English learning are more likely to control their efforts and regulate their learning process, which in turn contributes to the improvement of learning outcomes, supporting Hypotheses 2–4. The positive associations between English enjoyment, technology-based SRL strategies, and learning outcomes provide empirical evidence for the argument that motivational beliefs (e.g., enjoyment for learning) are major determinants of individual’s behavioral choices and learning achievement (Wigfield and Eccles, 2000). The results thus echo prior research findings which showed that students with high levels of learning interests tended to use more SRL strategies such as effort regulation, and get higher English test scores than their peers with lower enjoyment for English learning (Bai and Wang, 2020). Our result also lends support to Goetz et al.’s (2008) observation that academic enjoyment is strongly related to domain-specific academic self-concepts which, in turn, impact on students’ cognitions, behavior, and ultimate success in the academic domain. While the importance of enjoyment as an essential affective factor in L2 acquisition has been well acknowledged in the literature, its relationships with other learner variables have been under-researched particularly in an SRL context. This study thus adds to the limited existing research by showing that the role that enjoyment plays is two-dimensional in the current study as it has a strong relationship with SRL strategies and that this relationship is mediated by English language self-efficacy. The findings suggest a need to nurture students’ English language enjoyment and self-efficacy, which can be realized through providing diversified English learning activities (Bai and Wang, 2020), or through actively developing pedagogical practices that promote a positive self-concept and self-confidence among students so that they find the time spent there is a constant source of satisfaction, enjoyment, and self-efficacy (Dörnyei and Murphy, 2003).

The finding that a strong and positive relationship exists between English language self-efficacy and the technology-based SRL strategies leads us to concur with existing studies (e.g., Kim et al., 2015; Bai and Wang, 2020; Wang and Zhan, 2020) that postulate that students with higher level of self-efficacy tend to be more self-regulated than their peers with low self-efficacy profiles, reinforcing the consensus in the literature that positive beliefs about one’s learning capability tend to result in carrying out a learning task more readily, working harder, or persisting longer when learners encountered difficulties. Pedagogically, teachers can emphasize skill development and effective learning methods, as well as provide specific feedback and encouragement to enhance students’ confidence and sense of competence in authentic mastery experience (Liem et al., 2008). Particularly noteworthy is that among the four domains of English language self-efficacy examined in this study, English speaking competence appeared to be the most significant predictor of students’ use of a variety of technology-based SRL strategies. An important pedagogical implication of this result is that EFL teachers need to provide students with a fairly wide repertoire of speaking practice opportunities to help them maintain a positive belief about their English speaking competence, which in turn will likely be a vital driving force for students’ engagement in a range of SRL activities.

Although our study is the first to empirically investigate the associations among enjoyment, self-efficacy, SRL strategies in one model in relation to students’ learning achievement in the second language context, a number of limitations should be acknowledged and direction for future research needs to be provided. First, the present study adopted a cross-sectional design and the results only indicate associations or relationships between variables. As such, inferences about the causal relations among variables which require experimental design are not drawn. In future research that aims to examine causal relationships among these variables, longitudinal design that involves data collections in different points over a period of time should be adopted in order to draw inferences of causal relations. Second, the fact that participants in this study were from a few majors of one university may limit the generalizability of the findings. Future research needs to include students from different types of educational institutions and from a wider range of disciplines so as to gain more robust results concerning the dynamic relationships between enjoyment, self-efficacy, technology-assisted SRL engagement, and students’ learning achievement. Third, in this study, data were collected by means of self-reports, which might be susceptible to response bias as is the case with other survey-based investigations. While self-report has been widely used as a valid method for exploring student perceptions and feelings, future research can adopt additional objective measures, such as classroom observations, to minimize the limitations associated with self-reported data. Finally, our research model only explained 6.9% of the variance in EFL undergraduate students’ English learning outcomes, and a large portion of the variation remains uncaptured. Future research needs to explore potential variables that were not explored in the current model, such as students’ anxiety toward using technologies for English learning and the role of extrinsic motivation in driving students to learn English.

## Conclusion

This study contributes to the knowledge about Chinese EFL undergraduate students’ SRL strategies in technology-using conditions. The results of the study add to the literature that considers how technology-based SRL strategies are associated with students’ language learning achievement. From a theoretical perspective, our research extends SRL theories to technology-using language learning conditions, particularly with respect to the significant role of English enjoyment and English language self-efficacy, and in relation to students’ English learning outcomes. Pedagogically, awareness of the complex interrelationships among SRL strategies, English enjoyment, English language self-efficacy, and learning outcomes is helpful for educators to clearly understand what actually motivates and empowers students’ self-directed technology use for learning and the quality of this technology-based learning process. It is thus important for educators to create a pleasant and inspiring environment that empowers students in self-regulation of their technology-facilitated English learning practices so that they experience learning success and satisfaction inside and outside the classroom.

While this study is one of the pioneer studies conducted in an EFL context which addressed research gaps from previous SRL studies, it examined associations between technology-based SRL and students’ English learning outcomes in relation to only two learner variables, i.e., English language self-efficacy and English enjoyment. We understand the limitation of using cross-sectional data and we cannot draw cause-and-effect conclusions without a longitudinal design. We thus cautioned our readers of generalizing our results to cause-and-effect, especially the reciprocal relationship between enjoyment and self-efficacy (Malanchini et al., 2017). Future studies on technology-enhanced language learning can also investigate some variables that were not contained in the current model, such as students’ previous learning experiences and their willingness to use technology for language learning. As the items used in our TSRLSQ are newly proposed, further assessment with other populations is needed to provide evidence of external aspects of the construct validity of responses to the scale. In addition, this study relied solely on the use of self-reported data which is inherently subject to bias, and the findings therefore constitute only an overall picture of the participants’ experiences in terms of the measured variables. Future extensions of the research can make use of qualitative observational research to corroborate the statistical evidence reported in this study.

## Data Availability Statement

The raw data supporting the conclusions of this article will be made available by the authors, without undue reservation.

## Ethics Statement

The studies involving human participants were reviewed and approved by the University of Macau Ethics Assessment Committee. The patients/participants provided their written informed consent to participate in this study.

## Author Contributions

ZA and ZG conceived the idea and developed the materials. ZA carried out the data collection. ZA, SL, and ZG took the lead in writing the manuscript. CW, ZG, and HL provided critical feedback. All authors read and approved the final manuscript.

## Conflict of Interest

The authors declare that the research was conducted in the absence of any commercial or financial relationships that could be construed as a potential conflict of interest.

## Appendix A. Technology-Based Self-Regulated English Learning Strategies Questionnaire

### MRS (Motivational Regulation Strategies)

1. I select and use appropriate technological tools to improve the areas I’m weak in.
2. I use technologies outside the classroom to access authentic materials in English.
3. I search related materials online when I have difficulties in the process of studying English.
4. I seek opportunities through technological resources to practice my oral English.
5. I use technologies to help me sustain/enhance interest in learning English.
6. I use technologies (APPs or websites) to make the English learning task more interesting.
7. I use mobile devices to enhance my willingness to participate in English social events.
8. Sometimes I look through the visual and vivid courseware to arouse my interest in English learning.
9. When I feel bored with learning English, I adopt technological resources to decrease the boredom and increase the enjoyment.

### GS (Goal Setting and Learning Evaluation)

1. I listen to English radio broadcasts (e.g., VOA and BBC) to improve my English proficiency
2. At the beginning of the semester, I set technology-assisted English learning goals.
3. I often monitor my technology-assisted English learning progress.
4. I reflect on the effectiveness of using technologies for English learning.
5. I adjust my English learning plans in response to different technology-assisted learning activities.

### SS (Social Strategies)

1. I seek advice on how to use technologies effectively for English language learning.
2. I seek opportunities to talk with native English speakers through technological tools.
3. When I have problems in English learning, I ask my teacher for help through technological tools.
4. I share my problems with my classmates online so we can solve our problems together.

### TE (Technology-Based English Song and Movie Learning)

1. I “copy” useful words and expressions in English movies or programs.
2. I practice saying new expressions in English movies or programs to myself.
3. I listen to English songs to help me remember words.
4. I use technologies (e.g., English movies) to learn more about English and the culture.
5. I use technologies to connect English learning with my personal interest (e.g., playing English games, or listening and singing English songs).

### TV (Technology-Based Vocabulary Learning)

1. I use lexical apps to help me memorize new words.
2. I use online dictionaries to check English words.
3. I use technologies (e.g., vocabulary apps) to help me persist in my English learning goals.
